# Interrogating Epigenome toward Personalized Approach in Cutaneous Melanoma

**DOI:** 10.3390/jpm11090901

**Published:** 2021-09-09

**Authors:** Elena-Georgiana Dobre, Carolina Constantin, Marieta Costache, Monica Neagu

**Affiliations:** 1 Faculty of Biology, University of Bucharest, Splaiul Independentei 91–95, 050095 Bucharest, Romania; marieta.costache@bio.unibuc.ro (M.C.); neagu.monica@gmail.com (M.N.); 2 Immunology Department, “Victor Babes” National Institute of Pathology, 050096 Bucharest, Romania; caroconstantin@gmail.com; 3 Pathology Department, Colentina Clinical Hospital, 020125 Bucharest, Romania

**Keywords:** cutaneous melanoma, epigenetic regulation, inflammation, drug resistance, biomarkers, therapeutic targets, epigenetic therapy, immune response

## Abstract

Epigenetic alterations have emerged as essential contributors in the pathogenesis of various human diseases, including cutaneous melanoma (CM). Unlike genetic changes, epigenetic modifications are highly dynamic and reversible and thus easy to regulate. Here, we present a comprehensive review of the latest research findings on the role of genetic and epigenetic alterations in CM initiation and development. We believe that a better understanding of how aberrant DNA methylation and histone modifications, along with other molecular processes, affect the genesis and clinical behavior of CM can provide the clinical management of this disease a wide range of diagnostic and prognostic biomarkers, as well as potential therapeutic targets that can be used to prevent or abrogate drug resistance. We will also approach the modalities by which these epigenetic alterations can be used to customize the therapeutic algorithms in CM, the current status of epi-therapies, and the preliminary results of epigenetic and traditional combinatorial pharmacological approaches in this fatal disease.

## 1. Introduction

Cutaneous melanoma (CM) is an aggressive neoplasm that evolves from the malignant transformation of neural crest stem cell-derived melanocytes [1]. The etiology of melanoma is multifactorial, and the most prominent factors include genetic predisposition, light skin color, multiple naevi, and excessive exposure to UV [2]. Although less prevalent than other skin malignancies, CM accounts for more than 70% of skin cancer-related deaths. Its incidence, however, is steadily increasing in fair-skinned populations worldwide [3]. Detected in the early stages, CM is generally curable, with a 5-year overall survival (OS) rate for the localized disease of 95%. In contrast, stage IV patients carry a grim prognosis, and the 5-year OS rate drops to 25%. CM mortality is usually related to delayed diagnosis or tumor refractoriness to conventional therapies, all of which contribute to metastatic disease [4]. The remarkable progress made in recent years in deciphering CM biology has resulted in the development of several targeted therapies and immune-checkpoint inhibitors (ICIs), such as anti-programmed death (PD-1) and anti-cytotoxic T lymphocyte antigen (CTLA-4), that have truly revolutionized metastatic CM treatment. For instance, therapies targeting critical nodes in the mitogen-activated protein kinase (MAPK) pathway have significantly improved patient OS [5,6]. Furthermore, immune therapies with ICIs led to more durable results and even pathological complete response (pCR) in several patients [7,8]. However, a significant proportion of CM patients fail to respond to these therapies due to the quick emergence of resistance, suggesting that it is critical to gain a deeper understanding of this disease’s biology in order to improve its clinical management [9]. At present, CM diagnosis and prognosis rely on assessing clinicopathological variables that do not consider CM genomic and immune heterogeneity [10,11]. In CM, blood biomarkers are very few; S100 and melanoma inhibitory activity (MIA) are the only generally accepted biomarkers that can predict CM evolution and that can indicate therapy efficacy [12] Nevertheless, in tumor-free patients these biomarkers lack utility and additional biomarkers like those related to immune response can be used to monitor the disease dynamics [13]. Although still used in oncology as a general biomarker, serum lactate dehydrogenase (LDH) lacks specificity. An increase in serum LDH would indicate in late stages a high tumor burden, without providing any information on how to guide metastatic patient treatment [14]. Given the poor prognosis of advanced-stage CM, novel biomarkers are needed to assist in early diagnosis and prognosis and stratifying patients into different risk groups to optimize their therapeutic protocols [15].

**Figure 1:**
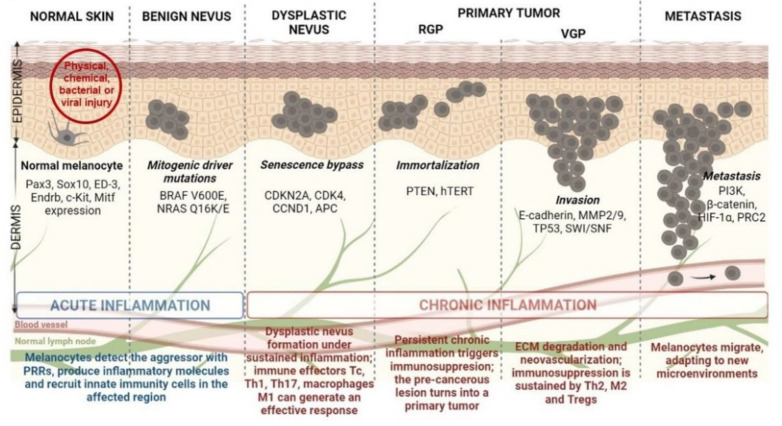
Schematic representation of CM development from healthy melanocyte to metastatic disease (the Clark model) under a pro-inflammatory milieu. Abbreviations: PRRs—pattern recognition receptors, Tc—cytotoxic T cells, Th1-T—helper type 1 cells, Tregs—regulatory T cells, Pax3—paired box gene 3, Sox10—SRY-box transcription factor 10, Endrb—endothelin receptor type B, c-Kit—human receptor tyrosine kinase c-kit, Mitf—microphthalmia-associated transcription factor, CDKN2A—cyclin dependent kinase inhibitor 2A, CDK4—cyclin dependent kinase 4, CCND1—cyclin D1, APC—adenomatous polyposis coli, PTEN—phosphatase and tensin homolog, hTERT—human telomerase reverse transcriptase, MMP2—matrix metalloproteinase 2, SWI/SNF—switch/sucrose non-fermentable chromatin remodeling complex, PI3K—phosphoinositide 3-kinase, HIF-1α—hypoxia-inducible factor-1α, PRC2—polycomb repressive complex 2.

The idealized model for CM development and progression is the Clark model. This model progressively describes the histopathological changes accompanying normal melanocytes’ linear progression to metastatic melanoma via a benign naevus [16] (Figure 1). Nevi are growth-arrested, clonal neoplasms of melanocytes, triggered by certain specific mutations in the MAPK pathway, usually BRAF V600E mutations [17]. Subsequently, the benign nevus evolves into a dysplastic nevus, which progresses through the primary tumor’s radial and vertical growth phases (RGP and VGP) and ultimately invades the dermis and regional lymph nodes, from which it metastasizes to distal sites [18]. However, current literature highlights that more than two-thirds of melanomas arise de novo in normal skin without requiring a nevus precursor [19,20]. Given that the annual risk of an individual nevus progressing into melanoma is estimated to be far less than one in 33,000 and the majority of CM patients lack atypical nevi, de novo genesis may be the main route of CM development [21,22]. Although nevus-associated melanomas correlate with favorable prognostic factors, the assessment of nevus as a biomarker in melanoma is still controversial due to certain unconventional models suggested for CM progression [21,23]. Finally, there is also an epigenetic progenitor model, which suggests that a polyclonal epigenetic disruption of tissue-specific (non-cancerous) stem/progenitor cells might act as a driving force of carcinogenesis. The subsequent accumulation of genetic and epigenetic alterations further enhances the tumorigenesis process [24,25] (Figure 1). Regardless of the controversy posed by CM progression, melanomagenesis is fueled by a pro-inflammatory environment. Multiple environmental agents can induce inflammation; however, UV radiation remains the most prominent aggressor, resulting in significant DNA damage and reactive oxygen species (ROS) generation [26]. Inflammation has acute and chronic stages that can shift the physiological balance towards skin regeneration or tumorigenesis, depending on their intensities [18,27]. If the inflammation is persistent and acquires chronic attributes, it may lead to a pre-cancerous lesion in the form of a dysplastic nevus. If detected by a fully functional immune system, this pre-cancerous lesion subsides to healing, and the melanocyte regains its physiological functions. However, if the entire process develops as a sustained chronic inflammation, it triggers a plethora of molecular and cellular networks shaping an immunosuppressive milieu that sustains skin tumorigenesis and further metastasis [18,28] (Figure 1).

Epigenetic alterations have emerged as essential contributors in the pathogenesis of various human diseases, including CM. Epigenetics is another layer of instructions apart from the genetic code that controls how genes are read and expressed, involving a change in the cell phenotype without changes in the genotype [29]. Epigenetic regulation is an umbrella term that encompasses several mechanisms such as DNA methylation, histone post-translational modifications (PTMs), nucleosome remodeling, histone variants, and RNA-mediated post-transcriptional regulation [30]. Influenced by lifestyle and environmental factors, epigenetic changes are highly dynamic and reversible and thus easy to regulate [31]. Given that these epigenetic alterations occur before the clinical diagnosis of CM, these molecular defects may serve as a foundation for developing novel diagnostic tools for early detection [32]. Furthermore, the discovery that distinct epigenetic signatures may associate with different disease subtypes highlights that these changes may also have prognostic applications in CM management [33,34]. Additionally, the study of epigenetic enzymes facilitates the design of novel treatment strategies that may help in delaying or reversing drug resistance, either as monotherapy or in combinations [35,36]. Of particular importance, observations that epigenetic regulators are often mutated in CM [37] and that mutant genes differ significantly between patients [38] suggest not only that the signaling pathways in these tumors are induced in a patient-dependent manner but also the need to implement personalized medicine in the clinical management of CM. Accordingly, once a certain abnormality is identified, it must be targeted with a specific treatment to reduce the side effects and maximize the therapeutic benefit of the patients [39]. Therefore, a better understanding of CM epigenetics will guide the precision medicine initiative in the field towards the identification of more specific diagnostic, prognostic, and predictive biomarkers, as well as more potent epigenetic inhibitors for the treatment of specific subtypes of CM.

We present herein both old and new evidence regarding the roles of DNA methylation and chromatin modifications in melanoma pathogenesis and discuss recent advances in investigating their translational potential as biomarkers and therapeutic targets.

## 2. Epigenetics: Another Layer of Information in Gene Expression Regulation

The epigenetics field focuses on the study of heritable alterations in gene expression with no underlying changes in the DNA sequence. Epigenetic regulation has been extensively reviewed in association with various human biological processes, such as embryogenesis, cell differentiation, X chromosome inactivation, and pathologies such as cancer [40,41]. The best-characterized epigenetic mechanisms are DNA methylation and histone modifications; however, the epigenetic scenario is much more complicated with new players and new mechanisms including non-coding RNA (ncRNA)-mediated regulation, histone variants, and ATP-dependent chromatin remodeling [42,43,44]. All these discrete but reversible modifications may orchestrate extensive changes in chromatin structure and conformation, interfering with the transcriptional machinery’s ability to access its target genes and promoters [45,46].

The epigenetic machinery carefully modifies the homeostatic balance between euchromatin and heterochromatin, which is essential to genomic stability [31]. The epigenetic players involved are divided into three classes: writers (generally enzymes that induce chemical modifications in histones and DNA), erasers (entities that erase these chemical signatures), and readers (enzymes that recognize and interpret various chemical modifications) [47]. Within the review, we will focus mainly on DNA methylation and histone modifications. These two types of epigenetic changes appear to influence each other in the deposition during mammalian development and carcinogenesis; histone methylation appears to direct DNA methylation patterns, while DNA methylation may serve as a model for establishing certain histone changes [32]. Moreover, in CM, DNA methylation is gaining increased importance in PD-related immune therapy [48], while histone alterations are associated with BRAF-targeted therapy [49].

In mammals, DNA methylation is an epigenetic signature that occurs at the 5’ position of the cytosine preceding a guanine nucleotide (denoted CpG, where *p* implies the phosphodiester bond between the two nucleosides) [50]. CpG-enriched regions, called ‘CpG islands’, are found in about 40% of mammalian gene promoters, making these particular regions prone to methylation [51]. Interestingly, methylation’s biological effects can vary widely depending on genomic location; thus, methylation in the promoter regions of genes is usually associated with transcriptional repression, while methylation in the gene body promotes transcription [31]. The enzymes that perform this modification are DNA methyltransferases (DNMTs), using S-adenosylmethionine (SAM) as a donor of methyl groups [52]. The human genome encodes at least four DNMTs: DNMT1, DNMT3A, DNMT3B, and DNMT3L [53]. DNMT1 is responsible for maintaining DNA methylation, while DNMT3A and DNMT3B catalyze the de novo synthesis of 5-mC [52,54]. Conversely, 5-mC methylation marks can be deleted by ten-eleven translocation (TET) proteins [55]. DNMTs may play critical roles in embryonic development and the intergenerational propagation of specific methylation patterns [56]; thus, any disorder in the functionality of these enzymes can have important physiological consequences [53]. DNMTs were recently shown to be involved in immune therapy resistance in CM, as further detailed in [57].

Histones, especially their N-terminal tails (containing an average of 15–30 residues), can undergo a plethora of PTMs, such as acetylation, methylation, phosphorylation, and more rarely, ubiquitination, sumoylation, and glycosylation [58]. The acetylation (ac) of histone lysines (K), is associated with the transition to an “open” conformation; this weakens DNA–histone interactions, increases DNA accessibility, and facilitates transcription. Acetylated lysine residues may also serve as binding platforms for transcription factors and other histone-modifying enzymes, such as bromodomains [59]. Another type of histone modification is methylation (me) of lysine and arginine (R) residues. Histone methylation can be present in several forms, so that the lysine residues may be mono-, di- or trimethylated, while the arginine residues may be mono- or di-methylated. Methylated histone residues are recognized by several protein domains such as plant homeodomain zinc fingers, Tudor domains, or WD40 repeats. While histone acetylation is usually associated with transcriptional activation, histone methylation has various functions, depending on the type of histone, the type of amino acid, the degree of modification, and another histone’s PTMs in the vicinity [60,61]. For example, in the promoter region, di- or tri-methylation of histone H3 at lysine 4 (H3K4me2, H3K4me3) is associated with transcriptional activation; in contrast, H3K27me3 and H3K9me3 are transcriptionally repressive marks [62]. Combinations of H3K4me1, H3K27me3, and H3K27ac marks can be used to distinguish active enhancers (H3K4me1 and H3K27ac positive) from inactive (H3K4me1 positive and H3K27ac negative) or poised (H3K4me1 and H3K27me3 positive) gene enhancers [63]. Interestingly, promoters of lineage-controlling developmental genes display a particular epigenetic signature that combines the activating H3K4me3 and the repressive H3K27me3 mark; these bivalent domains, which have also been reported in cancers, allow for rapid activation of developmental genes during embryogenesis, while maintaining repression in the absence of differentiation signals [64,65]. However, the balance between histone methylation and acetylation is tightly controlled by histone methyltransferases (HMTs)/demethylases (HDMs) and histone acetyltransferases (HATs)/histone deacetylases (HDACs), respectively [66]. Alterations in histone “writers” or “erasers” have been associated with many pathologies, including cancer [31,45,67]. Histone modifications can be associated in CM with therapy resistance [68] and/or with epithelial–mesenchymal transition (EMT) and metastasis [69].

## 3. CM Epigenetics

**Figure 2:**
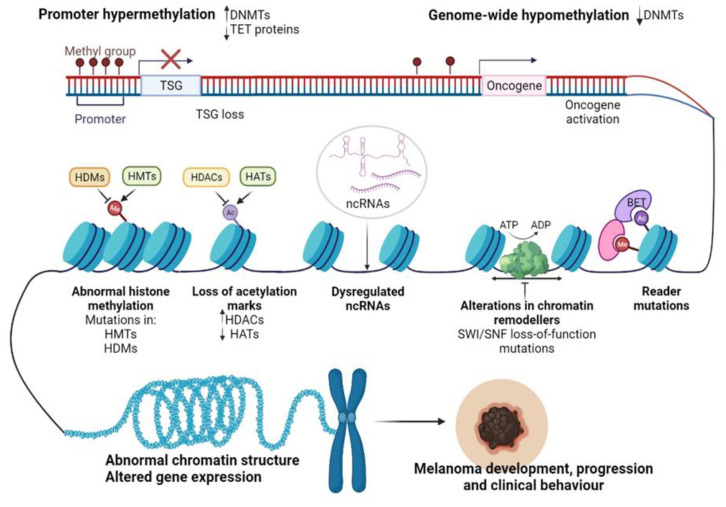
Epigenetic changes regulating CM progression and clinical behavior. Abbreviations: Me—methylation, Ac—acetylation, DNMTs—DNA methyltransferases, TET—ten-eleven-translocation enzyme, TSG—tumor suppressor gene, HDMs—histone demethylases, HMTs—histone methyltransferases, HDACs—histone deacetylases, HATs—histone acetyltransferases, SWI/SNF—switch/sucrose non-fermentable chromatin remodeling complex, BET—bromodomain and extra-terminal domain protein, ncRNAs—non-coding RNAs, ATP—adenosine triphosphate, ADP—adenosine diphosphate. Figure created with https://biorender.com/.

Recent advancements in next-generation sequencing (NGS) technologies and their coupling with chromatin-immunoprecipitation (CHIP), altogether RNA interference (RNAi) screening methods, and matrix-assisted laser desorption/ionization time-of-flight (MALDI-TOF) proteomic tools have allowed the dissection of the epigenome for various cancers [70]. In particular, for CM, epigenomic interrogation revealed aberrant DNA methylation in gene promoters [71], histone PTMs [72], alteration of epigenetic regulators [73], and dysregulated ncRNAs [74] (Figure 2). It seems that in CM these epigenetic alterations may allow melanocytes to overcome senescence and metastasize at a distance [75,76], support the immune escape of CM [77], but also the transcriptomic reprogramming of cancer cells to overcome the cancer therapy-induced apoptosis [78].

Inflammation and epigenetic alterations play pivotal roles in CM initiation and development. However, in recent years, considerable research efforts have been devoted to identifying a potential link between these processes in the context of cancer. Current literature confirms that these two phenomena are interconnected and mutually regulate each other [79,80]. Epigenetics can modulate tumor antigen presentation and immune cell functions, therefore impacting tumor development and clinical behavior [81]. Conversely, inflammation can induce epigenetic alterations in resident skin cells, promoting immune evasion and tumorigenesis [80]. We will focus herein mainly on inflammation-induced epigenetic changes in CM, as it is a less studied topic in the field. In the first instance, inflammation can disrupt epigenetic programs by altering the metabolic state of a cell [82]. Their activation determines alteration of immune cells’ metabolism and activated immune cells further disrupt the metabolic processes in neighboring tissues. Since the activity of many epigenetic enzymes depends on cellular metabolism intermediates, a dysfunctional metabolism will significantly impact the molecular processes within the cell [82]. For example, DNMTs and HMTs use SAM as a cofactor, while HDMs and TET proteins require α-ketoglutarate produced in the tricarboxylic acid cycle for their activity [83]. Moreover, it has been reported that increased production of cytokines, chemokines, and ROS, including hydrogen peroxides, can orchestrate dramatic epigenetic changes in resident epithelial cells. For instance, DNA damage triggered by ROS exposure can interfere with the ability of certain epigenetic regulators to bind to DNA, leading to abnormal DNA methylation patterns and altered gene expression [84]. Additionally, long-term production and accumulation of cytokines and ROS/reactive nitrogen species (RNS) have been correlated with the activation of STAT3 and NF-κB oncogenic pathways in epithelial cells [27,85]. In parallel, antigen-presenting cells (APCs) and cytokines can activate T cells, involving transcription factors that participate in their transcriptomic reprogramming, which includes epigenetic changes, among others [80]. Epigenetic alterations are usually pro-tumorigenic, facilitating the suppression of tumor suppressor genes and the activation of oncogenes. These reversible changes may also help tumors escape the immune response by reducing the expression of genes involved in the antigen processing and presentation or viral defense pathways. In line with these observations, myeloid-derived suppressor cells (MDSCs) may differentiate into tumor-associated macrophages (TAMs) or interfere with T cell activity, promoting tumorigenesis as transcription factors induce alternative transcriptional programs resulting in epigenetic alterations [80]. Here, we will describe the current status of knowledge regarding the roles of DNA methylation and chromatin modifications in CM as a problematic inflammatory malignancy.

## 4. Epigenetic Alterations Driving CM initiation and Progression

### 4.1. DNA Methylation in CM Development

Disruption of DNA methylation is a common event in cancer. Both focal hypermethylation at CpG islands and global hypomethylation are constant hallmarks of the cancer genome and often coexist in tumor cells, impacting tumor biology and behavior. As with other cancers, CM initiation and progression have been associated with loss of tumor suppressors and oncogene activation (Figure 2) [86].

Inactivation of tumor suppressor genes (TSGs) due to specific DNA methylation in the promoter regions was the first epigenetic alteration studied in CM more than 10 years ago [87] (Figure 1). So far, dozens of genes are known to be regulated by this mechanism. These genes appear to be involved in various signaling pathways, usually disrupted in CM, such as the phosphatidylinositol 3-kinase/protein kinase B (PI3K/Akt) and MAPK pathways, cell cycle, DNA repair, retinoblastoma (RB) and Wnt signaling [86]. Furthermore, it was also observed that three TSGs are frequently inactivated by methylation: RASSF1A (55%), RAR-β2 (70%), and MGMT (34%) can also be identified in the circulating tumor DNA of CM patients, which makes them useful diagnostic and prognostic biomarkers in the clinical setting [88,89]. In several cancers, but also in a significant proportion of melanomas, a gradual increase in DNA hypermethylation was observed along with tumor aggressiveness; this phenomenon, called CpG methylator phenotype (CIMP), was reported for the first time in colorectal cancers, a finding that highlighted a tight correlation between altered DNA methylation patterns and the clinical outcome of the affected patients. Tanemura at al. demonstrated that during tumor progression, several tumor-related genes and loci, including WIF1, SOCS1, RASSF1A, TFPI2, MINT17, and MINT31, gain methylation with advancing stages. These genes have been suggested to constitute CM’s CIMP [90]; however, recent research highlights that CIMP is usually associated with an NRAS-mutant phenotype, which is more aggressive than a non-NRAS-mutant tumor [91]. Therefore, future approaches should correlate CIMP with CM patients’ clinical outcomes and mutational profiles. This information would be essential for developing novel tools for prognosis and response to therapy in the clinical cohorts of melanoma patients.

Epigenetic silencing of tumor suppressor genes has been one of the best-studied phenomena in CM; however, although less studied, DNA hypomethylation is equally important in the initiation and development of CM. It was shown that in many cancers, hypomethylation contributes to tumor progression by inducing genome instability via the demethylation of transposons and pericentromeric repeats or the activation of certain oncogenes [92]. Figure 2 depicts hypomethylation mechanisms as a hallmark of melanomagenesis. LINE-1 elements are one of the most abundant classes of mobile DNAs within the human genome [93]. LINE-1 hypomethylation, detected in both tissues and plasma circulating DNA of melanoma patients seems to be a hallmark of the metastatic capacity of primary melanomas [94,95]. Other reports highlighted that LINE-1 hypomethylation may predict the OS in stage III CM patients [96,97]. In parallel, DNA hypomethylation has been described as one of the main mechanisms regulating the expression of cancer-testis-antigens (CTAs) in human melanomas [98]. CTAs are a specific group of tumor-associated antigens (TAAs) whose expression in normal tissues is generally restricted to the gametogenic tissues of the testis and fetal ovaries [99]; nonetheless, CTAs were found to be re-expressed via hypomethylation in CM, regulating vital cellular processes such as tumor cell division, differentiation, invasion and drug resistance [98,100,101,102].

Several studies have revealed that DNA methylome analysis may help discriminate between normal melanocytes, nevi, and melanomas. For instance, Fujiwara et al. identified several novel genes that were hypermethylated in melanomas compared to melanocytes, such as KRTCAP3, AGAP2, ZNF490, and TTC22, in addition to those previously documented, such as COL1A2, GPX3, and NPM2 [103]. Among those genes, they found that NPM2 showed distinct immunohistochemical (IHC) staining in normal melanocytes, whereas its expression was lost in CM samples [103]. Moreover, Gao et al. reported a diagnostic algorithm based on the methylation patterns of CLDN11, CDH11, and PPP1R3C genes that can differentiate between dysplastic nevi and primary melanomas with a specificity of 89% and a sensitivity of 67% [104]. Other reports highlighted that several methylation subgroups might be associated with different clinical characteristics of the disease, potentiating that the evaluation of DNA methylomes may have prognostic applications in CM. A study led by Lauss et al. revealed three methylation clusters: MS1, MS2, and MS3, which differ significantly in terms of promoter methylation, proliferation, and presence in immune cells [33]. The MS1 group has the highest methylation level, especially at CpG islands and poised promoters, enriched in polycomb repressive complex (PRC2) target genes. The MS1 subtype also showed an increased frequency of homologous deletions of CDKN2A and IDH1R132 hotspot mutations. The MS3 group had the lowest methylation levels, similar to peripheral blood leukocytes, and MS2 was intermediate. No correlations were identified between methylation clusters and clinicopathological variables or actionable mutations such as BRAF or NRAS. However, the tumors bearing MS1-signature were associated with the lower patient OS (20 months for MS1 vs. 60 months for MS3). Interestingly, genetic analysis revealed methylation clusters are associated with different biological and clinical behaviors. The MS1 subtype termed “proliferative” was associated with the upregulation of TP53, MDM2, CDK4, CDK6, CCND1, CCNE1, and E2F3, as well as epigenetic modifiers TET1, JARID1B, SWI/SNF chromatin remodelers, and DNMT3A; in contrast, the MS3 subtype harbored an “immune high” signature, possibly explaining the better survival of patients appending to the MS3 cluster [33]. Similarly, Yamamoto et al. stratified 51 CM into two risk groups based on the promoter region’s methylation status. The high-methylated subgroup was positively associated with a thicker tumor progression and hence a worse clinical prognosis. Among the 27 genes proposed to distinguish between the two subtypes, TFPI2 was the most frequently hypermethylated gene in the aggressive subtype [34]. In addition, altered methylation patterns of the homeobox D cluster were linked with melanoma metastasizing to the brain [105].

Further complicating this scenario, genome-wide mapping of CM revealed that 5 hmC levels are progressively lost during tumor progression from benign nevus to malignant melanoma, via IDH2 and TET family downregulation [106]. Elevated levels of 5 hmC were subsequently validated by IHC staining as predictors of metastasis-free survival and overall survival in CM patients [107]. Taken together, all this information supports the further development of 5-hmC IHC expression as a prognostic biomarker that can add some precision to the American Joint Committee on Cancer (AJCC) staging system [108].

### 4.2. Histone-Modifying Enzymes and PTMs in CM Development

In addition to the undeniable role of DNA methylation in melanomagenesis, histone PTMs mediated by several writers, readers and erasers may also alter some transcriptional processes closely associated with CM initiation and progression [109].

The involvement of histone modifications in CM development has been suggested since benign nevi, which usually carry the BRAF V600E oncogenic mutation, rarely become melanoma, as this conversion requires additional events. Yet, valuable insights on the importance of histone-modifying enzymes and PTMs in melanomagenesis were obtained using zebrafish melanoma models [110]. Patton et al. developed the first experimental model of BRAF V600E driven melanoma using a zebrafish model expressing BRAF V600E under the control of the mitfa promoter in a p53 loss-of-function background [111]. Only a fraction of zebrafish developed melanocytic tumors, highlighting the existence of additional molecular events operating in concert with genetic alterations in melanoma. To examine these processes in more detail, the researchers developed a triple transgenic zebrafish model (p53/BRAF/crestin: EGFP), in which the crestin/EGFP gene marks neural crest stem and progenitor cells, from which melanocytes originate [111]. Melanocytic tumors reported in zebrafish models re-expressed crestin-EGFP gene, suggesting that these cancer cells are maintaining their neural crest identity. Notably, they identified enrichment of H3K27ac marks in super-enhancers at the sox10 locus, a major regulator of neural crest formation and melanomagenesis, suggesting that epigenetic regulation of SOX10 is an important step in melanoma initiation [111]. Later on, Scahill at al. revealed that loss of kdm2aa, an orthologue of KDM2A, triggered the spontaneous formation of melanomas at a high frequency in zebrafish [112]. These tumors were generated independently of BRAF V600E and other melanoma-related mutations in oncogenes and tumor suppressors. Finally, gene expression analysis revealed altered levels of genes involved in DNA replication, translation, and chromatin regulation after kdm2aa silencing, confirming on an alternative pathway that histone methylation may have vital roles in melanomagenesis [112].

#### 4.2.1. Histone Modifications “Writers”

##### H3K4 Methyltransferases (KMT2D) 

One of the chromatin’s writer enzymes that has been identified to function aberrantly in melanomas is the KMT2D, also known as MLL2. KMT2D is associated with gene promoter and enhancer regions and catalyzes H3K4 monomethylation [113,114,115]. Several recent in vitro genetic screen studies have revealed important details regarding the roles of KMT2D in CM tumorigenesis [38,73]. By performing the first in vivo genetic screen with shRNA libraries targeting fundamental epigenetic players in CM, Bossi et al. observed multiple genes involved in melanomagenesis [38]. Among them, KMT2D orchestrates a migratory transcriptional program in NRAS melanomas. The authors also reported some interpatient heterogeneity in their study [38]. Interestingly, KMT2D silencing resulted in the inactivation of a subset of KMT2D-bound enhancers (reduced H3K4me1 and H3K27ac) and downregulation of MFGE8 and RPL39L cell motility genes. Notably, the closest genes to these enhancers were the KMT2D target genes, suggesting that KMT2D can deregulate enhancer activity to promote tumorigenesis [38]. Therefore, a better understanding of the roles of KMT2D in CM may help expand the number of biomarkers and druggable genes in the clinical management of CM patients.

After the first published results in 2016, KMT2D was reported as frequently mutated in a variety of solid and hematologic tumors, including melanomas (15%) [73]. Recently, Maitituoheti at al. identified KMT2D serving tumor suppressor roles in CM. In addition to KMT2D, the authors identified seven more epigenetic regulators in CM cell lines (KDM1A, APOBEC2, HDAC6, KMT2F, SETD4, KAT4, and KDM5B) whose loss accelerates CM tumor progression. Among them, KMT2D, KDM5B, KMT2F and KDM1A were mainly associated with H3K4 methylation. However, the most potent phenotypes were linked with mutations in KMT2D. To investigate CM genesis in more detail, the authors developed a genetically engineered mouse model (GEMM) based on conditional and melanocyte-specific ablation of KMT2D [73]. It has been further observed that H3K4me1-marked enhancer reprogramming by KMT2D loss is associated with a drastic alteration of the central metabolic pathways in the tumor cells. Furthermore, the authors observed a preferential dependence of glycolysis in deficient KMT2D tumors compared to WT cells, most likely to provide cancerous tumors’ energy and biomass needs. Interestingly, pharmacological inhibition of glycolysis and the IGF signaling pathway reduced the proliferation of KMT2D-deficient tumor cells in both murine models and human melanoma cell lines [73]. Thus, this study highlights exciting aspects of the biology of mutant KMT2D tumors and identifies new potential therapeutic vulnerabilities concerning them.

##### The Roles of H3K4 Methylation Marks

H3K4me1, H3K4me2 and H3K4me3 are generally associated with active gene transcription [116]. Although partially overlapping with H3K4me3 at the 5’-end level of transcribing genes, H3K4me2 decorates genomic regions independently of H3K4me3 [117]. H3Kme2’s role as a biomarker in the diagnosis and prognosis of CM patients has been suggested by several studies. For example, Uzdensky at al. found elevated levels of H3K4me2 in tumor samples compared to paired normal skin [118]. Later on, Kampilafkos at al. observed that H3K4me2 and H3K27me3 levels were lower in metastatic compared to primary melanomas and that combined IHC analysis of H3K4me2, H3K27me3, and EZH2 may help identify cancer cells with stem cell-like behaviors, particularly at the invasion front of CM [72]. This study highlighted that the combination of several genetic alterations may be more relevant for characterizing and predicting complex events such as metastasis and that all these epigenetic changes can be integrated as a code that can provide valuable information about the biology of melanocytic tumors.

H3K4me3 is a chromatin landmark of promoters of transcriptionally active genes or genes poised for activation in mammalian cells [116]. In particular, for CM, Cheng at al. observed that human metastatic tissues are highly heterogeneous in terms of H3K4me3 levels [115]. Further analysis showed that metastatic lesions that displayed low levels of H3K4me3 were associated with repressed genes in embryonic stem cells (ESCs) and PRC2-target genes. In contrast, elevated H3K4me3 levels correlated with interferon and inflammatory response genes [115]. However, Terranova at al. found that metastatic melanomas harbor exceptionally wide H3K4me3 domains [119]. These domains can span tens of thousands of kilobases, and appear to be involved in the regulation of several EMT transcription factors (POU3F2, SOX9, and PDGFRA) as well as melanocyte-specific master regulators (MITF, ZEB2, and TFAP2A) [120]. Terranova et al. finally highlighted that particular events such as BRAF or NRAS mutations may employ specific chromatin states (bivalent states and broad H3K4me3 domains) to orchestrate transcriptional changes unique to a genotype, suggesting that epigenetic mechanisms play important roles in regulating CM behaviors [119].

##### H3K27 Methyltransferases (EZH2)

Histone lysine methyltransferase EZH2, responsible for H3K27 trimethylation, was also found to be dysregulated during the development of human melanomas. EZH2 is the catalytic subunit of the PRC2 complex and appears overexpressed in various tumors, including melanoma [121]. PRC2 trimethylates H3K27, orchestrating the repression of transcriptional programs [121]. Particularly for melanoma, PRC2 levels have been reported to increase gradually over the progression from benign nevi to malignant melanomas, suggesting that this protein plays a key role in CM initiation and progression [72]. Moreover, EZH2 and H3K27me3 are overexpressed in highly invasive melanoma cells and metastatic melanomas, leading to TSGs inactivation [72,76,121,122]. Finally, other studies have revealed that increased EZH2 levels are associated with poor prognosis in CM patients [123].

Regarding the roles played by EZH2 in the pathogenesis of CM, several mechanisms by which it supports tumor growth and metastasis have been proposed. Some authors have shown that EZH2 expression is associated with the suppression of senescence in human melanoma cells. For example, Fan at al. have shown that EZH2 can support unlimited melanocyte proliferation by repressing CDKN1A, which is not mediated by H3K27me3 deposition [124]. Conversely, EZH2 silencing inhibits cell proliferation, restoring senescent phenotype and p21/CDKN1A expression in a p53-independent manner. It was further observed that depletion of EZH2 removes HDAC1 from the transcriptional start site of CDKN1A, resulting in increased H3 acetylation and transcriptional activation. These observations confirm the existence of a synergistic relationship between EZH2, as part of PRC2 and HDAC, in mediating the suppression of certain senescence-related genes in melanoma cells [124]. In parallel, De Donatis at al. showed that EZH2 oncogenic activation is mediated by the non-canonical NF-kB signaling pathway; interestingly, NF-kB2 silencing was associated with reconversion to the senescent phenotype, suggesting the pivotal roles of the NF-kB2/EZH2 axis in CM initiation and development [125]. Other studies have shown that induction of EZH2 in benign BrafV600E- or NrasQ61K-expressing melanocytes facilitates tumor metastasis and invasiveness by silencing genes relevant for cell surface organelle primary cilium integrity and by activating Wnt/β-catenin oncogenic signaling [77]. Finally, it is also worth mentioning that EZH2 gain-of-function mutations usually co-occur with BRAF V600E mutations in CM, promote aggressive cell morphologies and enhance melanoma tumor growth in vitro [126].

##### H3K9 Methyltransferases

Another histone-modifying enzyme involved in CM pathogenesis is bifurcated domain SUV39/SET 1 (SETDB1), which belongs to the SUV39 family of histone lysine methyltransferases [127]. This enzyme is involved in the trimethylation of H3K9, which is a specific signature of transcriptionally repressive chromatin [128].

In human melanoma samples, SETB1 often appears amplified in association with another histone methyltransferase, EHMT1 [129]. Subsequently, a positive association between SETB1 expression and several prognostic factors such as increased mitotic index, advanced Clark levels, and epidermal involvement in tissue biopsies of CM patients was also described [130]. A recent study, led by Orouji at al., revealed that the expression and amplification rate of SETDB1 may serve as an individual prognostic biomarker in CM, with increased levels of SETB1 protein being associated with metastasis and lower patient survival rates [131]. Compared to normal melanocytes, melanoma cells showed 8–13.9 times higher levels of SETDB1. Interestingly, they found that all those SETDB1 highly amplified human cell lines were BRAF V600E mutants [131]. Functional studies have shown that SETDB1 exerts its oncogenic effects in CM by modulating the expression of thrombospondin 1, a molecule known for its involvement in cell migration and invasiveness [131]. Surprisingly, it was found that SETDB1 regulates not only H3K9 methylation patterns but also H3K4me1 deposition, emphasizing that SETDB1’s involvement in tumorigenesis is much broader than previously thought. Furthermore, treatment with an H3K9me-specific histone methyltransferase inhibitor is highly effective in this context, leading to a considerable decrease in tumor cell viability. Interestingly, melanoma cells harboring low levels of SETDB1 were not affected by treatment with epigenetic inhibitors, underscoring SETDB1’s role as a valuable therapeutic target in CM [131].

#### 4.2.2. Histone Modifications “Readers”

Protein readers can recognize specific chromatin changes or combinations of PTMs and histone variants to further direct the transcriptional outcome [47]. Some of the best-studied families of chromatin readers are the bromodomain and extra-terminal domain (BET) family of adapter proteins (BRD2, BRD3, BRD4, and BRDT). BET proteins have an increased affinity for acetylated histone residues, enabling transcriptional activation by interaction with the transcriptional machinery [132]. Notably, the SWI/SNF complex, which uses energy from ATP hydrolysis to reshape the structure of chromatin, is also dependent on the presence of bromodomain-containing domains to be fully functional [133]. BET protein involvement in melanomagenesis was demonstrated by Segura at al. when it was shown that BRD2 and BRD4 are overexpressed in human melanoma cell lines and tissues, controlling the expression of certain genes involved in cell cycle progressions and survival [134]. Gene expression and IHC analysis of human tissue biopsies have confirmed higher levels of BRD2 and BRD4 in primary and metastatic tumors relative to melanocytes and nevi, suggesting that these BET proteins are involved in melanoma tumorigenesis [134]. Treatment with BET inhibitors (BETi) significantly reduced tumor growth and metastasis in both in vitro and in vivo models, and those effects were recapitulated by individual silencing of BRD4. Notably, the pharmacological capabilities of BETi have not been influenced by the mutational status of BRAF or NRAS, offering new promises for the treatment of patients that do not harbor actionable mutations [134]. Collectively, all these observations potentiate the pivotal role of the BRD4 protein in CM tumorigenesis, propelling it as a potential prognosis biomarker and therapeutic target in CM.

#### 4.2.3. Histone Modifications “Erasers”

##### Histone Deacetylases (HDACs)

One of the most important classes of chromatin erasers associated with CM pathogenesis is HDACs, which catalyze the deletion of acetyl groups from histone tails [135]. At least 18 mammalian HDACs were identified and subdivided into four main classes, depending on their location and functional characteristics. Given the significant differences reported between the acetylation patterns of benign nevi and the malignant tissues of patients diagnosed with CM, it was thought that aberrant histone deacetylation could play important roles in the pathobiology of CM [136]. Subsequent studies with patient-derived cell cultures have indeed shown that there is a loss of acetylation marks (H3K27Ac, H2BK5Ac, and H4K5Ac) and H3K4me2/3 during the transition from premalignant to the malignant phenotype, resulting in alterations of some essential signaling pathways in CM formation, including PI3K, interferon (IFN) -Ɣ, and TRAIL- and platelet-derived growth factor (PDGF) signaling [136].

Many other studies have shown that various HDACs are involved in skin tumorigenesis and can be exploited as biomarkers or therapeutic targets in melanomas. For instance, Wilmott et al. identified that increased expression of nuclear HDAC3 and cytoplasmic HDAC8 may serve as indicators of better prognosis in stage IV melanoma patients [35]. They also revealed an increase in HDAC8 levels in BRAF-mutant tumors [35]. Additionally, HDAC6 expression has recently been correlated with advanced stages and with an unfavorable prognosis [137]. In line with these observations, certain in vitro studies have shown that HDAC5 and HDAC6 are overexpressed in melanoma cell lines versus normal skin cells, being required for melanoma proliferation and metastasis through different signaling pathways [138]. Interestingly, HDAC6 inhibition inhibited tumor cell proliferation, and when knocked down cells were inoculated in animal models a decreased PD-L1 production and an augmented T-cell-mediated immune response was obtained [138].

Another epigenetic player investigated for its involvement in CM initiation is SIRT1 (class III HDAC proteins), which was found overexpressed in human melanoma cells and tissues in comparison to normal skin and melanocytes [139]. Further studies suggested that SIRT1 is upregulated in metastatic tumors compared to primary tumors, most likely due to its ability to support EMT programs via autophagic degradation of E-cadherin [140]. SIRT1, SIRT3 and SIRT6 were also proposed to support tumor growth in CM [141,142]. Interestingly, a recent study showed that dual inhibition of SIRT1 and SIRT3 mediated by 4′-bromo-resveratrol inhibits melanoma cell proliferation and growth [143]. Thus, all this information suggests that pro-tumorigenic sirtuins have not only the value of prognosis biomarkers but also of potential therapeutic targets in CM and that inhibition of multiple sirtuins may be a promising strategy for improving clinical management of CM.

##### Histone Demethylases (HDMs) 

The scientific progress made in recent years in deciphering the cancer epigenome has revealed the critical roles of histone H3 lysine 4 (H3K4) JARID1B/KDM5B demethylase in the tumorigenesis of various human tumors, including melanoma [144]. One of the pioneering studies in the field revealed that H3K4 demethylase JARID1B is overexpressed in melanocyte nevi and almost absent in melanoma samples [145]. However, subsequent studies have revealed increased heterogeneity of KDM5B in CM, and its expression levels have been documented to define distinct cellular states, even with antithetical effects on cellular tumor fate depending on the biological and clinical context [146]. KDM5B was found to mark a slow-cycling subpopulation of tumor cells which is essential for continuous tumor growth and resistance to therapy [147]. This population displays similar behaviors to that of cancer stem cells and give rise to a heterogeneous population of melanoma cells, being a major contributor to the increased heterogeneity observed in CM tumors [129]. Therefore, the assessment of KDM5B expression and H3K4 deposition patterns can provide valuable information about the clinical behavior of these tumors and may lead to more personalized therapies for CM patients.

H3K9me3 is an epigenetic mark of heterochromatin, which is often present on distal regions of genes [129]. H3K9 methyl groups may be erased by members of the lysine-specific histone demethylase (LSD) family. LSD1, often referred to as KDM1A, has the ability to demethylate histone 3 on lysine residues at position 4 (H3K4- gene promoter) and 9 (H3K9- distal) [129]. Interestingly, Yu et al. reported that oncogene-induced senescence of melanocytes relies on the deposition of H3K9me3 at the promoters of proliferation-related genes [75]. This is in accordance with their findings highlighting that benign naevi displayed increased senescence-associated H3K9me3 levels, with almost no detectable activity of H3 lysine 9 demethylases LSD1 and Jumonji Domain-Containing Protein 2D (JMJD2C/KDM4C), whilst human melanoma tissues generally harbored increased expression of LSD1 and JMJD2C and reduced H3K9me3 reactivity [75]. To gain a broader understanding of the molecular mechanisms that are involved in this process, the authors induced the expression of LSD1 and JMJD2C in mouse and zebrafish models. It was further shown that these two enzymes cooperated to overcome the oncogenic Ras G12V/BRAF V600E-induced senescence by preventing H3K9me3 deposition at E2F target gene promoters, which further augmented melanomagenesis [75]. Of note, targeted inhibition of LSD1 and JMJD2C demethylases restored cellular senescence and growth arrest, potentiating LSD1 and JMJD2C regulation as a potential anti-cancer therapeutic strategy [75].

## 5. Epigenetic Alterations Involved in CM Drug Resistance

### 5.1. Resistance to MAPK Inhibitors (MAPKi)

Genomic profiling of CMs revealed several actionable mutations in tumors that may be matched with targeted therapies. Recurrent driver alterations such as BRAF V600, NRAS, and NF-1 facilitated the design of BRAF inhibitors (BRAFi: vemurafenib and dabrafenib) and MEKi (trametinib, cobimetinib, and binimetinib) that have significantly improved patient OS [5,6]. Although 60–80% of BRAF-mutated CM patients respond well to targeted therapy, a significant proportion of them develop resistance, which results in life-threatening metastases and death [109,148,149]. The phenomenon of MAPKi resistance is complex and multifactorial and involves, among others, alterations of the BRAF V600E gene (amplification, aberrant splicing) [150,151,152], which leads to MAPK pathway hyperactivation, mutations that activate alternative survival pathways [153], modifications in apoptotic machinery [154], RTK hyperactivation [155,156], and the presence of slow-cycling populations [147], to which are added other MITFs, c-AMP, and NF-kB related mechanisms [9]. In CM, tumor refractoriness has been extensively linked with genetic alterations in many cancer-related genes; however, in some cases, the cause of the resistance appears to be non-genetic in nature [157]. Peculiarities such as the rapid kinetics and the transient nature of refractory phenotypes suggest the existence of an epigenetic basis for drug resistance in CM, pointing out that epigenetic remodeling is a fundamental feature of tumor development and adaptation to therapy [157,158]. Therefore, new therapeutic targets and therapies are critically necessary to improve the therapeutic management of CM. In this section, epigenetic alterations associated with MAPKi resistance and how they can be exploited in the future to become therapeutic targets and biomarkers in CM will be highlighted.

#### 5.1.1. DNA Methylation and MAPKi Resistance

Studies highlighting the involvement of DNA methylation in CM targeted therapy resistance are relatively few. Al Emran et al. reported DNMT3A, DNMT3B, and DNMT1 as differentially expressed in the BRAF V600E melanoma cells refractory to MAPKi, which resulted in low DNA methylation levels. However, genome-wide integrated epigenetic analyses revealed that altered histone methylation patterns, rather than DNA methylation, are involved in the transition from the normal state toward the resistant phenotype [159]. In parallel, Hugo at al. observed that drug resistance programs are associated with dramatic transcriptomic and methylomic alterations in MAPKi-treated CM patients. Transcriptomic analyses indicated dysregulated mRNA levels of LEF1, TAP1, CD8, and DUSP4 genes in MAPKi-resistant tumors, which correlated with differential methylation at CpG islands, suggesting the critical roles of DNA methylation in transcriptomic reprogramming of melanoma cells to support MAPKi resistance [160].

#### 5.1.2. Histone-Modifying Enzymes and PTMs Involved in MAPKi Resistance

One of the clinical observations that have postulated the link between epigenetic regulation and resistance to cancer therapy is the so-called “drug holiday” concept, which refers to intermittent treatment programs or treatment breaks. This strategy is often applied to delay the onset of resistance to therapy but is not potent on a genetically resistant phenotype [59]. Particularly for CM, it was observed that rechallenging patients with BRAFi after a free period of treatment and tumor progression resulted in a significant clinical response upon BRAFi and BRAF + MEKi treatments [161]. Recently, several studies have shown that the administration of a third-line BRAF-targeted therapy following first-line targeted therapy and second-line immunotherapy may be an effective strategy in CM metastatic patients [162,163]. Targeted therapy rechallenge in subjects who previously progressed on targeted therapies and immunotherapy was associated with a 2.7–5.9 month median progression-free survival (PFS), 9.3–19 month median OS and a 34–35% disease control rate. Notably, the time between treatment initiation and rechallenge did not seem to impact treatment responses [162,163].

Another aspect that advocates for epigenetically mediated drug-resistance phenotypes is that of slow-cycling cell populations, which appear to be endowed with reversible drug tolerance. One of the most important observations in this regard is that a very small fraction of cells can survive following exposure to drug concentrations 100-fold higher than IC_50_ [164]. These cells were found in a quiescent state and G1 arrest and continued to be viable in the presence of the drug. The induction of a “drug holiday”, however, resensitized these cells to initial therapy, potentiating the plasticity of the drug-tolerant phenotype of these cells. These refractory tumor populations showed an altered chromatin state, with elevated KDM5A expression levels and a dramatic depletion of H3K4me2/3 marks. Notably, RNA-mediated KDM5A silencing confirmed that this histone demethylase allows for the maintenance of a reversible drug-tolerant state in human melanoma cells [164]. The critical role of the KDM5B epigenetic regulator in the generation of cell subpopulations with distinct drug sensitivity profiles was recently confirmed in the study of Liu et al. [78]. They found in mouse melanomas two cell subpopulations, CD34+ and CD34−, endowed with the characteristics of stem and progenitor cells, which may differ considerably in their clinical behaviors. It was further observed that the CD34+ and CD34− subpopulations displaying the BRAFV600E mutation may respond differently to targeted BRAFi. Interestingly, KDM5B overexpression reprogrammed melanoma cells to a CD34−, more drug-tolerant, phenotype, while KDM5B loss shifted melanoma cells to a more BRAFi-responsive CD34+ state, potentiating the pivotal role of KDM5A in modulating intratumoral heterogeneity in CM [78]. Moreover, KDM5B, another H3K4 demethylase, has been observed to play similar roles in the responsiveness of CM tumors to targeted therapies [165].

Further complicating the drug-resistance scenario, several studies have highlighted those particularities of the tumor microenvironment such as hypoxia and nutrient starvation alongside genotoxic pressure exerted by drugs can give rise to induced drug-tolerant cells (IDTCs) rather than a selection of a pre-existing subpopulation. One of these studies revealed that continuous exposure of melanoma cells to sub-lethal BRAFi concentrations induced surviving cells to adopt a less differentiated state and become refractory to 20-fold higher BRAFi concentrations, as well as to other MEKi and platinum salts [166]. At the molecular level, it has been observed that IDTCs display exacerbated expression of drug efflux ATB-binding cassette transporters and melanoma stem cell markers and loss of differentiation markers such as melan-A and Tyrosinase, which are MITF-target genes. Depletion of histone marks H3K4me3, H3K27me3 alongside a remarkable increase in H3K9me3 was observed in IDTCs cells. The authors also reported an overexpression of several histone-modifying enzymes including the H3K27-specific demethylases, KDM6A, KDM6B, and the H3K4-specific demethylases, KDM1B, KDM5A, and KDM5B, in the IDTC states. Interestingly, as was observed for the KDM5A-enriched subpopulation, IDTCs regained their therapeutic sensitivity seven days after treatment interruption [166]. Hypoxic conditions and nutrient starvation were also associated with the transition to an H3K4me3low/H3K27me3low/H3K9me3high phenotype and the IDTCs generated in this manner exhibited increased refractoriness to BRAFi, suggesting an epigenetically regulated drug-independent stress response that allows cancer cells to cope with difficult environmental conditions [166]. All these observations enhance the role of tumor heterogeneity of CM as the main determinant of resistance to targeted therapies.

It is also well documented that epigenetic alterations can interfere with the MAPKi mechanism of action. MAPK inhibitors cause cellular apoptosis by adjusting the balance between members of the Bcl-2 family, more precisely, by inducing the pro-apoptotic factors Bim and Bmf and by reducing Mcl-1 expression [167,168]. In contrast, MAPKi-resistant melanoma cells showed overexpression of Mcl-1, concomitantly with Noxa downregulation, which counteracted the MAPKi-induced cell death [154,169]. Notably, inhibition of EZH2 expression has been associated with the release of apoptosis-inducing factor (AIFM1) from mitochondria and the induction of caspase-independent apoptosis in human melanoma cell lines [170]. Therefore, all this information suggests that using EZH2 inhibition in conjunction with MAPKi may be a promising strategy to combat the hurdle of drug resistance in CM. Moreover, it is well documented that the BET family of histone reader proteins also turns melanoma cells against apoptosis. There are at least two BET proteins in melanoma, in this case BRD2 and BRD4, that are documented to be overexpressed during melanoma progression. Interestingly, several studies have potentiated that inhibition of BET proteins is associated with an increase in BIM, which synergizes with the induction of BIM after MAPK inhibition, and that the combination of BETi and MAPKi may be an effective pharmacological approach in CM [134,171]. Equally exciting results were obtained by combining HDACi with BETi in CM cell lines, leading to deregulation of anti-apoptotic proteins and components of the AKT and Hippo/YAP signaling pathways [172].

Despite the remarkable progress made in understanding the biology of melanocytic tumors, drug resistance remains a major problem in CM therapeutic management. Epigenetic reprogramming has the potential to reshape the metabolic and signaling networks in cancers, facilitating the emergence of tumor cell subpopulations with distinct behavior and antigenic profile [173]. This intratumor heterogeneity drives new resistance mechanisms to escape the genotoxic pressure or the immune system, facilitating metastasis and disease relapse [109]. However, novel omics technologies such as single-cell analysis and clustered regularly interspaced short palindromic repeats (CRISPR)-associated protein 9 (Cas9) genome editing tool are expected to revolutionize CM research and partially solve the issue of intratumoral heterogeneity, either by screening for novel therapeutic targets or by functional genome/epigenome editing.

### 5.2. Resistance to Immunotherapy

The development of immunotherapy, which has transformed the management of metastatic tumors, has undoubtedly been fostered by a comprehensive understanding of the tumor microenvironment (TME) and its immunophenotype. Tumors can be reduced to two main compartments that are closely intertwined: the malignant cells and TME. TME is composed of a variety of stromal cells embedded in an extracellular matrix irrigated by a complex network of blood and lymphatic vessels [174]. The cells within the stromal compartment can include immune cells (macrophages, B lymphocytes and cytotoxic T lymphocytes (CTLs), natural killer cells (NKCs), neutrophils, and dendritic cells), mesenchymal cells (fibroblasts, myofibroblasts, cancer stem cells- CSCs, mesenchymal stem cells- MSCs, adipocytes, and endothelial cells), and MDSCs [175,176]. Moreover, in melanoma’s TME, TAMs are abundant and due to their pro-tumoral M2 phenotype these “tumor hijacked” cells sustain therapy resistance [15]. Stromal cells are in close communication with tumor cells and help them adapt to a changing microenvironment, survive, and replicate. As melanomas have a clear immune fate since immunosurveillance favors efficient tumor elimination and immunotolerance promotes tumor survival [175], therapy resistance has a clear immunological background. Additionally, it is well known that melanomas have an increased mutational rate and express a plethora of antigens, for example CTAs, which attracts immune cells that can eradicate the tumor or can be diverted towards pro-tumoral activity [177].

Natural immunosuppression has emerged as a physiological mechanism, but in TME immunosuppression usually interferes with CTL activity and functions [178,179]. Tumor cells mediate immunosuppression taking over inhibitory checkpoint proteins such as PD-1, T cell immunoglobulin and mucin domain 3 (TIM-3), lymphocyte activation gene 3 (LAG-3), and CTLA-4 expressed on the surface of T lymphocytes [180,181]. PD-1 and CTLA-4 bind to specific ligands such as PD-L1 and PD-L2, or CD80 and CD86, respectively, to negatively regulate T cell activity, leading to immune cell escape [181]. Therefore, the task of immunotherapy is much more challenging than it seems, as its goal is not to induce apoptosis but to modulate TME to induce a state of immunosurveillance that destroys cancer cells [109]. Anti-CTLA-4 antibody (Ipilimumab/Tramelimumab), approved for clinical management of CM more than five years ago [182] and followed closely by the approval of anti-PD1 (Nivolumab) [183], is the main immune therapeutical player that changed the fate of melanoma patients. Recently published data regarding the long-term therapy with individual and/or combined therapies in melanoma patients has shown that complete response is witnessed in 28% of patients and that there is still a great percentage of patients with incomplete response, patients that gain resistance and/or patients that due to immune-related adverse effect have to cease their immune therapy [184].

To date, the proposed mechanisms for CM resistance to immunotherapy are downregulation of MHC molecules, loss of antigenic expression, T-cell exhaustion, aberrant expression of PD-L1 in response to IFN-γ production by T cells, along with the altered expression of chemokines such as CCL3, CXCL1, and CCL4 [9]. Although it is well known that epigenetic regulation has critical roles in shaping the identity and differentiation of immune cells, the administration of epigenetic therapy should be done with caution in immunotherapy-resistant CMs, as these agents can affect other cells within TME in addition to tumor cells [59].

#### 5.2.1. DNA Methylation and Resistance to Immunotherapy

The discovery that the immune system can be harnessed to fight cancer and improve clinical outcomes in CM was recognized with a Nobel prize in 2018 [185]. Nevertheless, further studies drove research toward elucidating how DNA methylation can impact the function and activity of immune system components [57]. Earlier studies have shown that DNA methylation appears to be involved in regulating T cell differentiation and exhaustion [186,187], but also in modulating immune checkpoint genes [188], which are the main biomarkers for the response to immunotherapy.

Several authors have reported a mechanistic link between DNA methylation status and immune checkpoint gene expression that may have important predictive and monitoring implications for immunotherapy-treated CM patients. For instance, methylation of immune checkpoint CTLA4 has recently been associated with worse response and progression-free survival (PFS) in stage IV CM patients treated with ipilimumab [189]. The same study also highlighted an inverse correlation between CTLA4 promoter methylation and the presence of tumor-infiltrating lymphocytes (TILs), which play a critical role in tumor control and response to immunotherapy. Therefore, melanoma samples with a low level of methylation are likely to have an increased immune cell infiltration, and an increased number of TILs is an indicator of a good clinical response. However, no significant correlation was reported between CTLA4 promoter methylation and CTLA-4 protein expression, suggesting that the level of protein expression of CTLA4 cannot be used as a predictive biomarker in CM [189]. Other authors have shown that the pattern of PD-L1 methylation may also be suggestive of the response to immunotherapy. Briefly, it has been shown that the degree of DNA methylation of melanoma cells facilitates the stratification of CM patients into four subgroups based on the expression of PD-L1 and TILs and that this information may provide clues about the therapeutic response and survival rates of these CM patients [190,191]. Earlier studies have shown that highly responsive patients showed elevated levels of TILs and PD-L1, while the nonresponsive group displayed low levels of TILs and PD-L1 [190]. In the meantime, while clinical information has been gathered about this clear-cut PD-L1 high expression, efficient immune therapy has been shaken due to various newly discovered molecular mechanisms [192]. DNA methylome analysis of 52 stage III patients from TCGA revealed that low/absent PD-L1 expression is associated with high DNA methylation, differential expression of immune-related genes and worse survival [193]. In parallel, Micevic at al. confirmed that PD-L1 methylation regulates PD-L1 expression and identified for the first time the existence of methylated CpG loci at the PD-L1 promoter [194]. The authors further stratified melanomas into 2 groups based on PD-L1 status and observed that PD-L1 hypomethylation is associated with increased PD-L1 expression and superior OS in CM patients regardless of the diagnosed stages [194]. Moreover, studies on melanoma cells showed that treatment with the hypomethylating agent 5-azacytidine can orchestrate transcriptional derepression in hypermethylated PD-L1 tumors, leading to amplification of PD-L1 expression [194]. Finally, some other studies confirmed the epigenetic regulation of immune checkpoint gene LAG3 via DNA methylation in CM [195]. LAG3 is a molecule involved in blocking tumor cell proliferation and regulating the production of IFN-γ and TNFα cytokines. Interestingly, it has been shown that LAG3 promoter hypomethylation positively associates with increased levels of tumor-infiltrating immune cells and better PFS in CM patients [195].

Given that anti-PD-L1 and anti-PD-1 antibodies are the most extensively used immunotherapies in the clinical setting, and PD-L1 hypermethylation renders CM resistant to ICIs, applying DNMTi treatments appears a tempting strategy to reverse CM immunotherapy resistance. Interestingly, it has been noted that DNMTi hs the ability to activate endogenous retroviruses (ERVs) and virus defense-related pathways in melanoma cells [196]. In the TCGA, the expression levels of viral defense genes may help in stratifying primary samples from multiple tumors, including CM, into two risk groups where a high defense signature positively associates with improved OS and more durable clinical response. Moreover, combining anti-CTLA-4 with low doses of DNMTi proved to be an effective strategy in augmenting the immunotherapy efficiency in a mouse melanoma model [196]. Therefore, the use of immune checkpoint inhibitors in conjunction with DNMTi may be a promising strategy for maximizing the therapeutic benefit in CM patients. Certainly, we will soon find out more about the efficacy and safety of combining DNMTi with immunotherapy, given that ongoing clinical trials are studying the oral use of azacitidine with pembrolizumab in patients with metastatic CM (NCT02816021).

#### 5.2.2. Histone-Modifying Enzymes and PTMs Involved in Immunotherapy Resistance

To date, information on histone enzymes and PTMs involved in immunotherapy resistance are even vaguer than in the case of epigenetic regulation by DNA methylation. Several studies have suggested a mechanistic link between EZH2 activity and resistance to immunotherapy. For example, Zingg at al. observed that anti-CTLA-4 or IL-2 immunotherapy leads to TNFα amplification and T cell infiltration, resulting in EZH2 overexpression and loss of tumor control in melanoma mouse models [197]. Mechanistically, EZH2 catalyzes the deposition of H3K27me3 marks and the suppression of a plethora of immune-related genes. Notably, EZH2 inactivation reversed the drug-resistant phenotype and amplified the effects of anti-CTLA-4 and IL-2 immunotherapy in melanoma mouse models, thus blocking CM growth and dissemination [197]. Therefore, in this study, Zingg at al. have demonstrated not only that EZH2 expression is a valuable biomarker for monitoring the response to immunotherapy but can also be exploited as a therapeutic target to restore and enhance the effects of immunotherapy.

Additional studies have reinforced that the involvement of EZH2 in resistance to CM immunotherapy could be much broader. Tiffen at al. analyzed 471 cases of CM in the TCGA and found that 20% of patients displayed copy number amplifications and mRNA upregulation, along with activating mutations in EZH2 [198]. RNAseq analysis further showed that these alterations correlated with DNA hypermethylation and downregulation of certain genes involved in tumor suppression, antigen processing, and presentation pathways. Treatment with the EZH2 inhibitor GSK126 reversed the transcriptional silencing driven by EZH2 alterations in CM cells, suggesting that EZH2 inhibition is a promising pharmacological strategy for improving the therapeutic response in CM [198]. From these observations, it appears that there may be a close link between EZH2 and the activity of DNMTs in regulating tumor biological properties, including the response to immunotherapy in CM. It is well documented that the ATRX_DNMT3_DNMT3L (ADD) domain of DNMT3A may interact with several epigenetic players, such as SUV39H1 methyltransferases, HDAC1, and EZH2, among others [199]. Moreover, it was postulated that the activity of DNMTs is supported by EZH2, a well-known target of PI3K/Akt signaling, and that they cooperate in cancer pathogenesis [200]. In support of this idea is the observation that EZH2 and DNMTs are regulated by similar upstream signaling cascades, such as MEK/ERK and PI3K/Akt, and transcription factors such as NF-kB2 [57]. Last but not least, it appears that EZH2 can pre-mark genes for DNMTs methylation [201]. Therefore, although still in its infancy, the study of epigenetic mechanisms concerning the response to immunotherapy is expected to not only guide and revolutionize the treatment of refractory patients, but also to stratify them into risk groups to provide personalized therapeutic solutions.

## 6. Epigenetics-Based Therapies for CM

Given the critical roles of epigenetic alterations in CM development and its drug resistance, targeting or co-targeting these epigenetic events appears to be a promising strategy for improving the clinical condition of CM patients [109]. Although epigenetic biomarkers have not yet found their place in clinical practice, an impressive number of epigenetic drugs are constantly being developed and tested for their cytotoxicity and efficacy in clinical trials in various human cancers, including CM [89,202]. These epigenetic drugs include both general epigenetic inhibitors such as HDACi or DNMTi, but also more specific inhibitors targeting enhancer of zeste homolog 2 (EZH2i), bromodomain and extra-terminal domain proteins (BETi), or JMJD3 and JARID1B demethylases [203]. Table 1 summarizes the current status of those CM therapies (Table 1).

DNA methylation and histone acetylation were the first and most extensively studied epigenetic alterations in cancer. The progress made in understanding them led to the development of DNA methyltransferase inhibitors (DNMTIs) and histone deacetylase inhibitors (HDACi), which constitute the first generation of epigenetic inhibitors [39]. These first-generation epigenetic inhibitors were tested in clinical trials either alone or combined with other therapeutic agents; notably, these molecules showed limited selectivity and stability, increased cytotoxicity, and low efficiency in solid tumors especially when employed as a single therapy [235]. Their clinical use is currently restricted to hematological malignancies: myelodysplastic syndromes and leukemias. The low efficacy of epi-therapies in solid tumors compared to blood cancers is still poorly understood. One possible explanation may be that these agents reach their therapeutic concentrations more efficiently in blood cancers so that their short life may not affect their activities as it may do in solid tumors [236]. Another explanation refers to the fact that solid and hematological tumors differ considerably in terms of cell differentiation and epigenetic plasticity, with solid tumors originating from a more terminally differentiated state that is much more difficult to be transcriptomically reprogrammed [39].

The introduction of the second-generation of epi-drugs, which included certain DNMTi (such as zebularine and guadecitabine) and HDACi (belinostat, panobinostat, hydroxamic acid, tucidinostat, and valproic acid) has brought considerable advantages over its predecessors. These compounds have improved pharmacological properties, fewer side effects, and amplified selectivity, their targets being key drivers or pivotal regulators of tumor growth [202]. Despite the scientific efforts devoted to its development, the second generation of epigenetic drugs also showed reduced efficacy when administered as monotherapy [237]. This called for the development of the third generation of epi-therapies, which include, among others, histone methyltransferase inhibitors (HMTi), histone demethylase inhibitors (HDMi), enhancer of zest homolog 2 inhibitors (EZH2i), and bromodomain and extra-terminal domain inhibitors (BETi) [39]. The development of the third generation of epi-drugs took into account the principles of precision medicine, in which the existence of a high degree of selectivity is a supreme desideratum [39]. The development of the third generation of epi-drugs revealed that epigenetic factors that can write, erase or read epigenetic marks are usually protein complexes, emphasizing that a better understanding of the epigenetic regulators’ interactome may help to design more effective and selective epi-therapies [238]. In this section we will review the current status of epigenetic therapies used either as single agents or in combination with conventional approaches in CM.

### 6.1. Epigenetic Drugs as Monotherapies in CM

#### 6.1.1. DNMTi

DNMTis are potent antineoplastic compounds able to reverse the DNA hypermethylation status of TSGs. Depending on their mode of action, DNMTIs can be divided into two classes: cytosine analogue inhibitors and non-nucleotide analogue inhibitors [239].

Cytosine analogues such as azacytidine, decitabine, zebularine, guadecitabine (SGI-110), fazarabine, and pseudois cytidine, may replace C-5 of cytosine with N-5 into the DNA or RNA backbone, leading to DNMTs degradation, DNA damage, and subsequently, apoptosis [239]. Although they gained FDA approval for the treatment of hematologic malignancies, azacitidine and decitabine showed disappointing results in CM and other cancer patients with solid tumors when employed as monotherapy in Phase I/II clinical trials [240]. The clinical benefits seen in patients with solid tumors exposed to high doses of azacytidine and decitabine are offset by severe adverse effects, such as hematological toxicity [203]. The lowest dose at which decitabine was shown to be effective in treating a solid tumor was 50 mg/m^2^ but the reported side effects were severe [241]. Interestingly, the incidence of hematotoxicity may be reduced if these nucleoside analogues are administered by hepatic arterial infusion and not via the intravenous route [242]. Moreover, it seems that low doses of DNMTis (~20 mg/m^2^) can minimize toxicity and side effects while making tumor cells more sensitive to immunotherapeutic or chemotherapeutic agents [242]. Further clinical research revealed that cytosine analogs can induce ERVs and CTA expression in cancer patients so that cancer cells end up expressing a plethora of neoantigens that can be targeted by immunotherapy [243]. Moreover, it has been reported that DNMTi exposure can induce the activation of transposable elements such as Alu or LINEs, leading to a state of viral mimicry in which treated tumor cells translate the induced expression as caused by an exogenous viral infection, ultimately triggering an innate immune response [203]. Combining DNMTi with chemotherapy is another intensively investigated strategy to improve the clinical management of CM patients. A Phase I/II study combining decitabine with temozolomide in patients with metastatic melanoma showed promising results in terms of efficiency and safety profile (NCT00715793) [244]. The study reported that administration of decitabine in combination with temozolomide re-sensitizes CM patients to temozolomide by dual modulation of DNA repair machinery due to depletion of DNA repair protein O6-methylguanine-DNA-methyltransferase (MGMT) and subsequent induction of DNA mismatch repair (MMR) pathway in temozolomide-refractory melanoma cells [244].

Other cytosine analogues that are gaining considerable research interest for clinical use in CM are guadecitabine and zebularine. Although these next-generation DNMTis act similarly with azacytidine and decitabine, they have a longer half-life and better bioavailability when compared to their predecessors [237,239]. Guadecitabine has been tested in over 30 clinical trials in cancers, including certain phase III trials (https://clinicaltrials.gov). In particular, for CM, there are no clinical trials in which guadecitabine is tested as monotherapy, there are only two ongoing clinical trials, but in which it is administered in conjunction with immunotherapy (NCT02608437, NCT04250246). Zebularine is another second-generation DNMTi that acts by sequestering DNMTs after incorporation into DNA, interfering with their catalytic activities [245]. Preclinical studies in CM highlighted that zebularine treatments can reverse the mechanisms developed by the tumor to get rid of anti-tumoral immunity, priming it for the action of immunotherapies. That is, zebularine allows for the re-expression of intercellular adhesion molecule-1 (ICAM-1) on tumor endothelial cells, which results in restored leukocyte-endothelial cell adhesion and enhanced leukocyte infiltration [216]. Zebularine remains to be further evaluated in clinical trials in CM.

Another class of DNMTi is the class of non-nucleotide analogue inhibitors. This class encompasses small molecules that block the interaction of DNMTs with target sequences either by binding to the catalytic site of DNMTs or by binding to CpG-enriched sequences; however, the antineoplastic effects of non-nucleotide analog inhibitors are inferior to those of cytosine analog inhibitors. They include several compounds such as hydralazine, epigallocatechin gallate (EGCG), and disulfiram [239]. Disulfiram is one of the best-studied non-nucleotide analog inhibitors in cancers, and implicitly in CM. In numerous in vitro experimental studies, disulfiram and its metabolites were extremely potent in blocking tumor growth and inducing apoptosis in tumor cells, but with the disadvantage of strong cytotoxic effects. However, no clinical reports attest to a measurable efficacy of disulfiram as a mono-therapeutic agent against solid tumors to date, most likely due to the low circulating bioavailability of this compound [246]. Due to the potent antiapoptotic activity of disulfiram in tumor cells, it remains a promising approach when administered in conjunction with other pharmacological approaches such as chemotherapy, targeted therapy, radiotherapy, or immunotherapy. However, to minimize the cytotoxic effects and increase the therapeutic benefit of cancer and CM patients, extensive testing of disulfiram is needed in experimental studies and clinical combinatorial trials [246].

#### 6.1.2. HDACi

HDACis are a class of compounds capable of rectifying the aberrant acetylation status of histone and non-histone proteins in cancers to orchestrate TSG activation. In addition, cancer cells display an increased sensitivity to HDACi-induced apoptosis, making those epigenetic agents promising anti-cancer strategies [239]. The FDA has approved four HDACis for use in cancer patients. Three of these compounds are the hydroxamic acids vorinostat, belinostat, and panobinostat, which have been described as non-specific HDAC inhibitors targeting all classical HDACs (classes I, II, and IV). In contrast, the fourth HDACi that gained FDA approval, romidepsin, also known as FK228 or depsipeptide, has been reported to act specifically on Class I HDACs [247]. Other HDACis that have been tested in vitro or are in current clinical trials are well characterized by the scientific literature [203]. Proposed mechanisms of action for HDACis include cell cycle arrest by p53-dependent or -independent induction of the p21CIP/WAF1 axis, downregulation of oncogenes, enhanced ROS production, autophagy induction, as well as suppression of genes involved in cell survival and EMT programs [245,248].

Several HDACis, including both specific (class I HDAC inhibitor entinostat) and non-specific agents (panobinostat), have recently been tested in phase I/II clinical trials in metastatic CM patients; yet, they have shown reduced efficacy and poor tolerability as monotherapies [249,250]. The most common side effects associated with HDAC inhibition are hematotoxicity, fatigue, nausea, and hyperglycemia [229]. Valproic acid (VA) administration also led to disappointing results in CM clinical cohorts [227]. This was mainly because valproic acid requires a long period, up to several weeks, to reach full dose in most patients, making it almost ineffective in the case of aggressive and rapidly dividing CM tumors. VA was linked to serious adverse effects, such as grade 3 neurological toxicity and intracerebral hemorrhage [227]. In a phase II clinical trial, vorinostat (suberoyl-anilide-hydroxamic acid: SAHA) demonstrated some early responses in patients with advanced CM; however, for the majority of these patients, the disease state was stable, and vorinostat did not meet its primary endpoint of response [251]. As expected, vorinostat therapy was associated with significant side effects, such as lymphopenia, fatigue, and nausea [251]. Surprisingly, quisinostat, an HDACi with increased affinity for class I and II HDACs, showed strong antineoplastic activities and an acceptable safety profile in phase I clinical study of metastatic melanoma patients, calling for further investigation of its clinical efficiency and tolerability in different doses and combinations [252].

In recent years, considerable attention has been paid to sulforaphane (SFN), a natural compound found in cruciferous vegetables, such as broccoli, cauliflower, cabbage, and Brussels sprouts. Being a natural compound, SFN brings with it the advantage of being well-tolerated in human subjects [245]. Additionally, in CM preclinical studies, SFN consumption was linked with the inhibition of UV-induced inflammation and tumorigenesis [253,254]. It was further reported that SFN, through its inhibition of HDACs properties, can regulate apoptotic programs and cell survival pathways to induce cell arrest and apoptosis in melanoma cells [255]. Other mechanisms by which SFN can control tumor growth and evolution in CM cells are by downregulating the metastasis-promoting enzymes MMP-9 and sulfatase-2 [256,257]. However, a pilot study with 17 patients with at least two atypical nevi and a prior history of melanoma revealed that SFN treatment is relevant for chemoprevention in this clinical condition [228]. SFN treatment at 200 μmol daily for 28 days resulted in a significant reduction of the proinflammatory cytokines IL-10 (CXCL10), MCP-1 (CCL-2), MIG (CXCL9), and MIP-1β, and overexpression of tumor suppressor decorin. These encouraging results support that further testing of SFN should be performed in phase II clinical trials at higher doses and over a longer period, ideally accompanied by morphological, histopathological, and molecular analyses of excised tumors [228]. Other HDACis such as trichostatin A (TSA) and suberic bishydroxamate (SBHA) are currently in the preclinical development phase for CM treatment [229].

#### 6.1.3. Next-Generation Epigenetic Agents

Novel epigenetic targets identified in cancers have given rise to the next generation of epigenetic therapies that include enhancer of zest homolog 2 inhibitors (EZH2i), bromodomain, and extra-terminal domain inhibitors (BETi), inhibitors of the demethylases JMJD3 and JARID1B, as well as many other compounds [202]. The observation that EZH2 is often overexpressed in cancers led to the development of certain small molecule agents targeting this histone methyltransferase such as EPZ-6438 (tazemetostat), GSK2816126, and CPI-1205 [239,258]; these drugs are currently in early clinical studies reporting clinical responses with acceptable tolerability in many cancers [258,259]. EZH2 is also amplified in CM, and increased levels have been linked with aggressive disease and poor prognosis [77]. Notably, the use of EZH2i in the treatment of wild-type and mutant melanoma cell lines led to a reduction in cell proliferation and growth, legitimating EZH2 as a veritable target in melanoma cells [170]. Another study highlighted that EZH2 depletion may interfere with cancer cell proliferation both in vitro and in vivo via the induction of the p21/CDKN1A-mediated senescence [124]. Moreover, conditional ablation of EZH2 in a melanoma mouse model impaired tumor growth and attenuated metastasis without affecting the functionality of normal melanocytes [76]. These effects were recapitulated by pharmacological inhibition of EZH2, suggesting that EZH2 inhibitors are promising therapeutic approaches for the treatment of CM patients [76].

Another class of epi-therapies that is gaining considerable research interest in cancers is that of BET inhibitors (BETi). BETis exert their anti-neoplastic activities in tumor cells by preventing BETs-acetylated histones interaction [239]. So far, several BETis, such as thienodiazepine JQ1, I-BET151/GSK1210151A, I-BET762/GSK525762, ABBV-075, PLX51107, ODM-207, and ZEN003694 have shown encouraging clinical outcomes with tolerable toxicity and increased efficiency in various tumor types [260,261]. BRD4 is a notorious BET family member that is consistently reported to be amplified or overexpressed in human melanoma lines and primary tumors [262]. Notably, one study suggested that BETi treatments significantly affected melanoma cell proliferation in vitro and tumor growth and metastasis in vivo due to the downregulation of genes involved in cell cycle progression (SKP2, ERK1, and c-MYC) and concomitant accumulation of cyclin-dependent kinase inhibitors (p21 and p27). These effects were also reported upon individual silencing of BRD4. However, the efficiency of BETi was not affected by the BRAF or NRAS mutational status, suggesting that BRD4 inhibitors may be a promising approach for the treatment of CM patients that are not eligible for targeted therapy [134]. NHWD-870, a novel BRD4 inhibitor that suppresses the secretion of macrophage colony-stimulating factor (CSF)-1 by tumor cells and disrupts the BRD4/HIF-1α axis, is currently under preclinical investigation for the treatment of CM [233]. Another BETi, I-BET151, triggered the selective inhibition of the NF-κB signaling pathway in melanoma cells, regulating genes involved in inflammation (VEGF, CCL-20) and cell cycle progression (CDK6) and suppressing the production of IL-6 and IL-8 via BRD2 displacement [263]. PLX51107, a next-generation BETi, impaired tumor growth to differing degrees in BRAF V600E syngeneic mouse models, effects that were linked to the influx of TAMs. Tumors that were poorly responsive to BET inhibition displayed an increased influx of pro-tumoral macrophages and the addition of PLX3397 resulted in improved response rates to PLX51107, offering a novel combination therapy for metastatic CM patients [234]. In another study, PLX51107 slowed the growth of mouse BRAF V600E melanoma tumors by inducing the CD8^+^ T cell-mediated anti-tumor effects; moreover, PLX51107 proved to be an effective second-line therapy for CM tumors that harbored resistance to PD1/PDL1 checkpoint inhibition [264]. While certain BETis have recently started to be evaluated in phase I clinical trials in CM patients [232], novel pharmacological inhibitors continue to be tailored to target other classes of epigenetic enzymes, such as JMJD3 and JARID1B histone demethylases [265,266]. Therefore, the continuous enrichment of the anti-cancer armamentarium with next-generation therapies and their exploration either alone or in conjunction with traditional therapies is expected to positively impact CM therapeutic management and open up new opportunities for precision medicine in those patients.

### 6.2. Combinatorial Therapies in CM

Several lines of evidence support that, in addition to their potential use as single agents, epigenetic drugs may be extremely potent in sensitizing tumor cells to other anti-cancer therapies or may help in circumventing the major hurdle of acquired drug resistance [68,203,267]. Chemotherapy, targeted therapy, and immunotherapy are the major pillars of the anti-cancer treatment arsenal in CM. In this section, we review the current literature by which epi-drugs may regulate the sensitivity of cancer cells to targeted therapies and immunotherapies, two of the most used therapies in CM clinical management.

#### 6.2.1. Combinations with Targeted Therapy 

Several research groups have shown that the administration of epigenetic inhibitors, such as HDACs, BETi, or DNMTi in combination with BRAFi and/or MEKi can reverse acquired resistance to targeted therapy. For instance, the use of BRAFi encorafenib in combination with HDACi panobinostat decreases PI3K oncogenic pathway activity and increases the expression levels of pro-apoptotic proteins BIM and NADPH oxidase activator (NOXA) in CM cell lines with acquired BRAFi resistance [268]. In parallel, it has been reported that BETi JQ1 treatment can sensitize vemurafenib-resistant melanoma cells to BRAFi by modulating the histone acetylation patterns of the P-gp gene in the promoter region to prevent drug efflux [267]. Additionally, combining BET and HDAC inhibitors has strong synergistic effects in the induction of apoptosis and suppression of Akt and Yap signaling in melanoma cells, even in those with acquired resistance to BRAFi [172]. In addition, the combination of vemurafenib and decitabine has shown increased efficacy and tolerability in BRAF V600E metastatic tumors in a Phase I clinical trial (NCT01876641) [212]. Although this study was prematurely terminated due to loss of funding, 3/14 patients achieved a complete response, 3/14 had a partial response, and 5/14 had stable disease. The highest response rate was reported in clinical cohorts that utilized low-dose, long-term decitabine, emphasizing that future studies should focus on long-term use of decitabine at the lowest dose of 0.1 mg/kg [212]. At the moment, a phase II clinical trial is testing the efficacy and safety of tazemetostat in combination with dual BRAF/MEK inhibition in patients with BRAF-mutated metastatic melanoma who progressed on prior BRAF/MEKi therapy (NCT04557956).

#### 6.2.2. Combinations with Immunotherapy 

**Figure 3:**
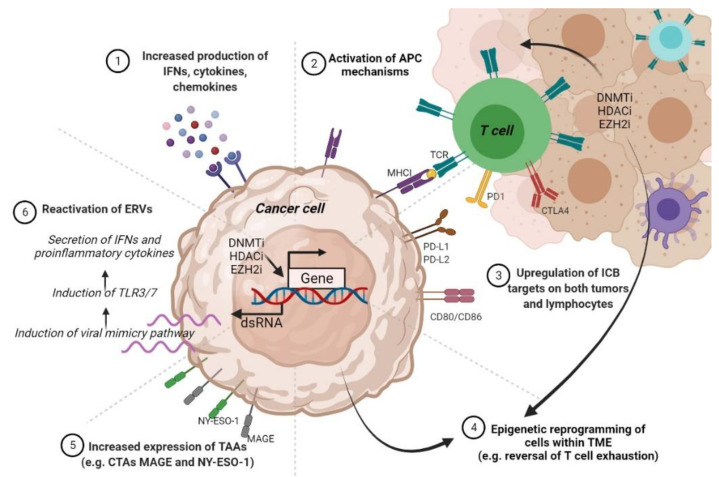
The proposed mechanisms by which epigenetic agents may regulate the immune response in cancers. Abbreviations: IFNs—interferons; APC—antigen-presenting cell; MHCI—major histocompatibility complex I; TCR—T-cell receptor; PD-1—programmed cell death protein 1; CTLA4—cytotoxic T-lymphocyte-associated protein 4; ICB—immune checkpoint blockade; PD-L1—programmed death-ligand 1; CD80—cluster of differentiation 80; TME—tumor microenvironment; TAAs—tumor-associated antigens; CTAs—cancer testis antigens; MAGE—human melanoma antigen; NY-ESO-1—New York esophageal squamous cell carcinoma 1; TLR3—Toll-like receptor; dsRNA—double-stranded RNA.

Several other reports highlighted that combining epigenetic therapies with immunotherapeutics may result in considerable clinical benefits in cancer patients. This is possible due to the ability of epigenetic therapies to modulate the immune response in tumors [269]. The mechanisms by which these epi-therapies exert their activity are not fully elucidated, but it has been suggested that they may involve reactivation of ERVs, upregulation of tumor-surface antigens, stimulation of antigen-presenting mechanisms, activation of IFN response pathways, transcriptomic reprogramming of TME cells, or induction of immune checkpoint blockade targets such as PD-1/PD-L1 on both tumor cells and lymphocytes alongside reversal of T cell exhaustion [79]. The mechanisms underlying these actions are depicted in Figure 3.

A bourgeoning body of evidence suggests that despite their reduced anti-cancer activities, DNMTis have the potential to increase the immunogenicity and immune recognition of CM cells [270]. Pioneering studies in this field have highlighted that DNMTi hypomethylating agent decitabine may orchestrate re-expression of HLA class I antigens on melanoma cells, which enables tumor cell recognition by MAGE-specific cytotoxic T lymphocytes and their further elimination [271]. Since the studied melanoma cell line was obtained from a metastatic lesion of a nonresponding patient undergoing MAGE-3.A1 T-cell-based peptide immunotherapy, the authors postulated that hypermethylation-induced loss of HLA class I expression may be the cause of the impaired response to vaccination; however, this study highlights for the first time that DNMTis should be tested for their efficiency on reversing the acquired resistance to immunotherapy in CM [271]. Other preclinical studies have shown that DNMTi may trigger the activation of ERVs, which are normally transcriptionally silenced, leading to the activation of a type I IFN response and cytotoxic T cell recruitment into the TME [196,272]. DNMTi treatments were also linked with CTA induction in melanoma cells, with important pharmacological applications in CM management, as the homogeneous expression of a therapeutic target in neoplastic cells is a prerequisite to effectively target tumors by vaccination-induced CTA-directed immune response [273]. MAGE-A3 and NYESO-1-based vaccines are currently being tested in clinical trials for the regression of CM (Li at al. 2020). Therefore, epigenetic regulation may be harnessed to broaden the eligibility and benefits of vaccine-based therapies in CM patients, but also to tailor specific immunotherapies for each patient [273].

Currently, two clinical trials evaluating the safety and efficiency of DNMTi combined with immunotherapy are underway in CM. Preliminary results of a phase II study of azacitidine in combination with pembrolizumab in patients with metastatic melanoma (NCT02816021) revealed that although the combination is relatively well tolerated, it is potent only in PD-1-naïve patients (55% ORR) [274]. However, these observations remain to be further confirmed in larger clinical cohorts and correlated with the molecular characteristics of tumor biopsies. Another phase I clinical trial evaluating guadecitabine with anti-CTLA-4 showed promising tumor immunomodulatory and anti-cancer activities in CM metastatic patients; briefly, the use of DNMTi led to the induction of HLA class I molecules and IFNƔ signaling pathways and increased tumor infiltration by CD8^+^ T cells, rendering those tumors more sensitive to immunotherapy [214].

Recent studies have provided evidence that the efficiency of immunotherapy may also be potentiated in cancers when used in combination with HDACis. HDACis are known to increase MHC class I and II expression on the cell surface or regulate PD-L1 and PD-L2 expression on cancer cells, thereby rendering the tumor vulnerable to T-cell mediated immune responses [275,276]. Furthermore, it is well documented that HDACis may be involved in TME reprogramming to deactivate immunosuppressive cells such as MDSCs and increase cytotoxic T cell activity within the TME [277]. Interestingly, in a melanoma mouse model, HDAC6 inhibitor Nexturastat A given in conjunction with anti-PD-1 antibodies significantly impaired tumor growth by causing a decrease in the anti-inflammatory phenotype of macrophages and increased infiltration of CD8^+^ T cells and NK cells into the TME [278]. Moreover, upon exposure to the HDACi entinostat in combination with azacytidine, the syngeneic mouse melanoma models displayed improved rates of response to both anti-PD-1 and anti-CTLA-4 immunotherapies, and the tumors were eradicated in 80% of the experimental animals [279]. Functional analysis revealed that the primary targets of the epigenetic drugs were the MDSCs, which exert immunosuppressive effects in tumors [279]. Similarly, mocetinostat decreases immunosuppressive immune cell phenotypes while augmenting anti-tumor phenotypes in both preclinical and clinical settings, providing a rationale for the use of these agents in conjunction with ICIs in CM [225]. The promising results of treatment regimens incorporating immunotherapy and HDACi have led to the recent initiation of several clinical trials in CM. Preliminary results from a phase I clinical trial evaluating the combination of pembrolizumab and HDACi entinostat demonstrated a favorable response in patients with immune checkpoint inhibitor-resistant CM and acceptable safety (NCT02437136) [224]. Notably, for one patient with a confirmed partial response, transition from a PD-L1 negative phenotype (pretreatment tumor biopsy) to a PD-L1 positive state (post-treatment biopsy) was reported, as a hallmark of increased sensitivity to immunotherapy [224]. In contrast, in another phase I clinical trial, panobinostat showed limited efficiency when added to standard ipilimumab therapy in CM metastatic patients (NCT02032810) [222]. Other drug combinations such as that of anti-PD-L1 antibody nivolumab with tinostamustine, an alkylating HDACi (NCT03903458) are now under clinical investigation in refractory, locally advanced, or metastatic CM patients (Table 1).

Finally, several studies that highlight the combination of EZH2i and MAPKi or immunotherapy show an enhanced apoptosis and increased tumor control in CM cell lines and mouse models [197,198]. There is also the possibility of using EZH2i in conjunction with DNMTi and/or HDAC inhibitors as these agents have demonstrated the ability to upregulate immune response pathways in many solid cancers, including CM [57,235].

## 7. Discussion

Notorious for its increased unpredictable behaviors, distant metastatic patterns, increased inflammatory status, and intrinsic resistance to therapy, CM is an unsolved clinical and social issue [280]. The remarkable progress made in recent years in deciphering CM biology has resulted in the development of several targeted therapies and immune checkpoint inhibitors that have truly revolutionized the treatment of metastatic CM. For instance, therapies targeting nodes in the MAPK pathway have greatly improved OS; furthermore, immune therapies with immune-checkpoint modulators have led to more durable results and even pCR in several patients [192]. Despite these promising results, targeted and immune therapies’ effectiveness is often limited by the emergence of drug resistance. In CM, tumor refractoriness has been extensively linked with mutations in genes regulating drug efflux mechanisms, apoptotic machinery, DNA damage repair, cancer stemness, as well as many other biological processes [280,281,282,283]; however, for a significant proportion of CM, the cause of the resistance appears to be non-genetic. Peculiarities such as the rapid kinetics and the transient nature of refractory phenotypes suggest the existence of an epigenetic basis for drug resistance in CM, pointing out that epigenetic regulation is a fundamental feature of tumor development and adaptation to therapy [157]. Accordingly, epigenetic alterations are currently being investigated as potential biomarkers and are being envisioned as promising therapeutic targets for CM clinical management [284].

We revised how epigenetic mechanisms such as DNA methylation and histone modifications are fine-tuning gene expression programs in CM, thus influencing almost all the biological properties of these tumors. Distinct epigenetic signatures show promise for assisting in distinguishing between benign and malignant lesions, as well as between certain disease subtypes histologically classified or evaluated into blood circulation [34,103,285]. Moreover, altered epigenetic patterns have been shown to contribute to the acquisition of drug resistant phenotypes in CM and some of these modifications seem to have important prognostic and predictive applications [189,195]. Given the critical roles of epigenetic alterations in CM development and drug resistance, but also their reversible nature, targeting or co-targeting these epigenetic events appears to be a promising strategy for improving the clinical condition of CM patients. Although epigenetic biomarkers have not yet found their place in clinical practice, an impressive number of epigenetic drugs are constantly being developed and tested for their cytotoxicity and efficacy in clinical trials in CM [203].

There are several generations of epigenetic drugs. The first two generations of epi-therapies include DNMTis and HDACis, some of which have already gained FDA approval for the treatment of blood cancers [202]. Notably, these earliest generations of epigenome-targeted therapies were tailored according to the “one size fits all” principle, showing poor efficiency and selectivity and increased toxicity in CM patients [39]. However, clinical responses achieved with certain third-generation epigenetic agents designed according to precision medicine paradigms have provided new hope in the treatment of solid cancers [286,287]. Therefore, epi-drug development should follow a more personalized approach, with further identification of robust predictive biomarker selection and subsequent validation of this strategy in clinical studies [39]. Current literature highlights that, besides their use as monotherapy, epigenetic drugs may synergize with other anti-cancer compounds and reverse therapy resistance in both preclinical and clinical settings [236]. The administration of certain epigenetic drugs before chemotherapy or targeted therapy can be used for priming cancer cells to be more sensitive to these approaches, as epigenetic drugs can induce chromatin decompaction, making it more accessible to antineoplastic compounds [203]. A phase I clinical study (NCT01876641) has already shown that vemurafenib is more effective in CM patients when given in combination with decitabine in low doses. In line with these observations, the preclinical assessment reported activity of the combination and an increased potential in delaying the development of acquired resistance [212]. Moreover, co-administration of two epigenetic agents (such as DNMTi and HDACi) with different mechanisms of action may allow these compounds to increase each other’s efficiency and overcome the issue of CM drug resistance [279]. Another rationale for using DNMTi in conjunction with HDACi is that such a combination may be extremely potent in stimulating the immune system to fight against tumors [39]. Finally, combinations of epigenetic agents with immunotherapy are currently tested in CM in clinical trials, holding promise to enhance antitumor immune responses or to reverse acquired resistance to ICIs. Even though most clinical studies have simple designs with both agents used simultaneously and continuously, epi-drugs and immunotherapy combinations are generally well tolerated in human subjects, in contrast to regimens incorporating targeted therapies and immunotherapies [39]. However, long-term use of certain epigenetic agents may have detrimental effects on antitumor immune response; for instance, prolonged use of BETi was linked with T cell depletion [288]. Therefore, to use these epigenetic drugs in immuno-oncology, more scientific effort is required to understand how the dose and scheduling of these therapies modulate the immune response and adverse effects in the clinical setting of melanoma management.

## Figures and Tables

**Table 1:** Overview of the most common epigenetic inhibitors and their current status in CM ^1^.

Class	Agent	Mechanism of Action	Development Stage	Combination	References
DNMTi	Azacitidine (Vidaza©)	Incorporation into DNA, covalently linking the DNMTs, leading to DNMTs exhaustion and DNA damage	Phase I/II clinical trials	- (NCT02223052)	[204]
				Pembrolizumab (NCT02816021)	[205]
				Carboplatin + Avelumab (ACTRN12618000053224)	[206]
	Decitabine (Dacogen©)		Phase I/II clinical trials	- (NCT00002980)	[207]
				TCR-engineered T-cell immunotherapy (NCT02650986)	[208]
				Decitabine, Temozolomide and Panobinostat (NCT00925132)	[209]
				Temozolomide (NCT00715793)	[210]
				Vemurafenib (NCT01876641)	[211]
				Vemurafenib + Cobimetinib (NCT01876641)	[212]
	Guadecitabine (SGI-110)		Phase I/II clinical trials	Ipilimumab (NCT02608437)	[213,214]
				Nivolumab + ipilimumab (NCT04250246)	[215]
	Zebularine		Preclinical	-	[216]
	Disulfiram (Antabuse©)	Prevent the interaction of DNMTs with their target sequences either by binding to the catalytic site of DNMTs or by binding to CpG-enriched sequences	Phase I clinical trials	- (NCT00256230)	[217]
				Arsenic Trioxide (NCT00571116)	[218]
HDACi	Vorinostat/Suberoylanilide hydroxamic acid (SAHA/Zolinza©)	Targeting class I, II and IV HDACs	Phase I/II clinical trials	- (NCT02836548)	[219]
	Domatinostat (4SC-202)	Targeting class I HDACs	Phase I clinical trials	Nivolumab + Ipilimumab (NCT04133948)	[220]
	Panobinostat (LBH589)	Inhibition of class I, II, and IV enzymes	Phase I clinical trials	- (NCT01065467)	[221]
				Ipilimumab (NCT02032810)	[222]
	Romidepsin (Despipeptide/FR901228)	Targeting class I HDACs	Phase II clinical trials	- (NCT00104884)	[223]
	Entinostat (SNDX-275/MS-275)	Inhibition of class I HDACs	Phase II clinical trials	Pembrolizumab (NCT02437136)	[224]
	Mocetinostat (DB11830)	Targeting class I HDACs	Phase I clinical trials	Ipilimumab (NCT03565406)	[225]
	Tinostamustine	Targeting all the classical HDACs	Phase I clinical trials	Nivolumab (NCT03903458)	[226]
	Valproic acid (Depakote©)	Inhibition of class I and II enzymes	Phase I clinical trials	Chemoimmunotherapy	[227]
	Sulforaphane	Regulation of inflammatory and cell survival pathways	Pilot clinical studies	- (NCT01568996)	[228]
	Trichostatin A (TSA)	Induction of cell cycle arrest and apoptotic pathways	Preclinical	-	[229]
	Dacinostat (LAQ824)	Regulation of cell cycle and apoptosis	Preclinical	-	[229]
	Suberic bishydroxamate (SBHA)	Induction of apoptosis	Preclinical	-	[229]
EZH2i	Tazemetostat	Targeting EZH2 activity	Phase I/II clinical studies	Dabrafenib trametinib (NCT04557956)	[230]
	CPI-1205	Targeting EZH2 activity	Phase I clinical studies	Ipilimumab (NCT03525795)	[231]
BETi	ODM-207	Preventing BETs-acetylated histones interaction	Phase I clinical studies	- (NCT03035591)	[232]
	NHWD-870	Preventing BETs-acetylated histones interaction	Preclinical	-	[233]
	PLX51107	Preventing BETs-acetylated histones interaction, regulation of TME	Preclinical	PLX3397	[234]

^1^ (-) none; (+) combination therapy.

## Data Availability

Data sharing not applicable.

## Abbreviations

MAPK—mitogen-activated protein kinase; OS—overall survival; ICIs—immune-checkpoint inhibitors; PD-1—anti-programmed death; CTLA-4—anti-cytotoxic T lymphocyte antigen; LDH—serum lactate dehydrogenase; S100—S-100 protein; MIA—melanoma inhibitory activity; PTMs—post-translational modifications; EZH2—enhancer of zeste 2 polycomb repressive complex 2 subunit; SWI/SNF—switch/sucrose non-fermentable chromatin remodeling complex; pCR—pathological complete response; CDKN2A—cyclin-dependent kinase inhibitor 2; CDK4—cyclin dependent kinase 4; RASSF1A—ras association domain family member; RAR-β2—retinoic acid receptor-beta; WIF-1—WNT inhibitory factor 1; Socs1—suppressor of cytokine signaling 1; TFPI2—tissue factor pathway inhibitor 2; KRTCAP3—keratinocyte associated protein 3; STAT3—signal transducer and activator of transcription 3; NF-κB—nuclear factor kappa B; AGAP2—Arf-GAP with GTPase, ANK repeat and PH domain-containing protein 2; ZNF490—zinc finger protein 490; TTC22—tetratricopeptide repeat protein 22; COL1A2—collagen type I alpha 2 chain; GPX3—glutathione peroxidase 3; CLDN11—claudin 11; CDH11—cadherin 11; PPP1R3C—protein phosphatase 1 regulatory subunit 3C; NPM2—nucleophosmin/nucleoplasmin 2; CCND1—cyclin D1; CCNE1—cyclin E1; E2F3—E2F transcription factor 3; JARID1B—jumonji AT-rich interactive domain 1B; TFPI2—tissue factor pathway inhibitor 2; IDH1/2—isocitrate dehydrogenase ½; KDM1A—lysine demethylase 1A; APOBEC2—apolipoprotein B mRNA editing enzyme catalytic subunit 2; SETD4—SET domain containing 4; KDM5B—lysine demethylase 5B; POU3F2—POU class 3 homeobox 2; SOX9—SRY-box transcription factor 9; PDGFRA—Platelet derived growth factor receptor alpha; MITF—melanocyte inducing transcription factor; ZEB2—zinc finger E-box binding homeobox 2; TFAP2A—transcription factor AP-2 alpha; EHMT1—euchromatic histone lysine methyltransferase 1; RTKs—receptor tyrosine kinases; LEF1—lymphoid enhancer binding factor 1; TAP1—transporter associated with antigen processing 1; CD8—cluster of differentiation 8; DUSP4—dual specificity phosphatase 4; CCL3—chemokine (C-C motif) ligand 3; CXCL1—C-X-C motif chemokine ligand 1; TNFα—tumor necrosis factor alpha; p21CIP/WAF1—cyclin-dependent kinase inhibitor 1A.
